# Next-Generation Contraceptive Intravaginal Ring: Comparison of Etonogestrel and Ethinyl Estradiol In Vitro and In Vivo Release from 3D-Printed Intravaginal Ring and NuvaRing

**DOI:** 10.3390/pharmaceutics16081030

**Published:** 2024-08-02

**Authors:** Isabella C. Young, Allison L. Thorson, Mackenzie L. Cottrell, Craig Sykes, Amanda P. Schauer, Rani S. Sellers, Rima Janusziewicz, Kathleen L. Vincent, Soumya Rahima Benhabbour

**Affiliations:** 1 Division of Pharmacoengineering and Molecular Pharmaceutics, UNC Eshelman School of Pharmacy, University of North Carolina at Chapel Hill, Chapel Hill, NC 27599, USA; isabellayoung2015@gmail.com; 2 Joint Department of Biomedical Engineering, North Carolina State University and The University of North Carolina at Chapel Hill, Chapel Hill, NC 27599, USA; allythor@live.unc.edu (A.L.T.); januszrx@email.unc.edu (R.J.); 3 Division of Pharmacotherapy and Experimental Therapeutics, UNC Eshelman School of Pharmacy, University of North Carolina at Chapel Hill, Chapel Hill, NC 27599, USA; mlcottre@email.unc.edu (M.L.C.); craig_sykes@unc.edu (C.S.); aps5@email.unc.edu (A.P.S.); 4 Department of Pathology and Laboratory Medicine, University of North Carolina School of Medicine, Chapel Hill, NC 27599, USA; 5 Department of Obstetrics and Gynecology, University of Texas Medical Branch, Galveston, TX 77555, USA

**Keywords:** contraception, drug delivery, intravaginal rings, 3D printing

## Abstract

Intravaginal rings (IVRs) represent a well-established, woman-controlled and sustained vaginal drug delivery system suitable for a wide range of applications. Here, we sought to investigate the differences in etonogestrel (ENG) and ethinyl estradiol (EE) release from a 3D-printed IVR utilizing continuous liquid interface production (CLIP™) (referred to as CLIP_LOW_ for low drug loading and CLIP_HIGH_ IVRs for high drug loading) and NuvaRing, a commercially available injection molded IVR. We conducted in vitro release studies in simulated vaginal fluid to compare the release of ENG and EE from CLIP_LOW_ IVRs and NuvaRing. CLIP_LOW_ IVRs had a similar hormone dose to NuvaRing and exhibited slightly slower ENG release and greater EE release in vitro compared to NuvaRing. When administered to female sheep, NuvaRing demonstrated greater ENG/EE levels in plasma, vaginal tissue and vaginal fluids compared to CLIP_LOW_ IVR despite similar drug loadings. Leveraging observed hormones levels in sheep from NuvaRing as an effective contraceptive benchmark, we developed a long-acting CLIP_HIGH_ IVR with increased ENG and EE doses that demonstrated systemic and local hormone levels greater than the NuvaRing for 90 days in sheep. No signs of toxicity were noted regarding general health, colposcopy, or histological analysis in sheep after CLIP_HIGH_ IVR administration. Our results provided (1) a comparison of ENG and EE release between a 3D-printed IVR and NuvaRing in vitro and in vivo, (2) a preclinical pharmacokinetic benchmark for vaginally delivered ENG and EE and (3) the generation of a 90-day CLIP IVR that will be utilized in future work to support the development of a long-acting ENG/EE IVR combined with an antiretroviral for the prevention of HIV and unplanned pregnancy.

## 1. Introduction

Intravaginal rings (IVRs) are torus-shaped polymeric devices dispersed with active pharmaceutical ingredients (APIs) designed to promote controlled drug delivery via the vaginal tract. IVRs as a delivery system exhibit many advantages as they capitalize on highly vascularized vaginal tissue promoting drug uptake and avoid first-pass metabolism, thus increasing bioavailability, and are suitable for various applications [1,2]. Furthermore, IVRs can have high acceptability from end-users due to their long-acting ability, easy insertion and removal and being woman-controlled (i.e., with no assistance needed for insertion and removal). Additionally, IVRs are well-established drug delivery devices and have been in development for over five decades. The first IVR in clinical development appeared in the 1970s [3], and since then there have been many IVRs marketed for contraception and hormone replacement therapy (NuvaRing^®^, Annovera^®^, Estring^®^, Femring^®^, Progering^®^ and Fertiring^®^). Furthermore, in 2021, a dapivirine releasing IVR was recommended by the World Health Organization for HIV prevention [4,5], which was the first IVR preventative product for protection against HIV. Additionally, there are many IVRs in preclinical and clinical development as multipurpose prevention technologies (MPTs), which address two or more sexual or reproductive health indications in a single IVR, such as prevention against HIV, other sexually transmitted infections and unplanned pregnancy [1,6,7,8,9,10,11,12].

Despite the advances driven by IVRs in the women’s sexual and reproductive health landscape, all IVRs that are commercially available and most IVRs in development are manufactured by hot melt extrusion and injection molding (IM). With this conventional manufacturing method, raw materials (e.g., API, polymer, excipients) are fed into the IM machine, heated and mixed thoroughly to produce a homogenous melted mixture. The melted mixture can be (1) injected into an IVR mold to generate simple drug-loaded matrix rings or (2) co-extruded to form rods, cut to a desired length and thermally welded into the final shape, which is typically seen with reservoir-style or segmented IVRs [13]. Although this fabrication method is high-throughput and well established, it involves high heat and pressure, permitting the use of only API and material choices that can withstand harsh conditions. Furthermore, IVR molds are restricted to consist of a solid cross-section, preventing 100% drug release and thus requiring IVRs to be overloaded with APIs to achieve therapeutic efficacy [14]. 

Fabricating IVRs with additive manufacturing or 3D-printing can overcome IM limitations by implementing IVR design [1]. Three-dimensional-printed products are produced using computer aided design (CAD), which enables the opportunity to incorporate designs within IVRs during fabrication. This allows for the solid cross-section to be eliminated, which will alter its surface area and drug diffusion distance to promote controlled and complete API release [15,16]. Utilizing 3D-printing for IVR fabrication can promote an unlimited design space to fine-tune API release to achieve precise drug delivery targets. Additionally, 3D-printing can reduce API exposure to high heat and pressures that are commonly associated with IM manufacturing, allowing for more sensitive APIs to be considered for vaginal delivery through IVRs and expanding their use for other indications. Specifically in the contraceptive landscape, 3D-printed IVRs can incorporate more sensitive contraceptives, such as non-hormonal contraceptives that may not be amenable to an IM manufacturing process. 

Only a few studies have been conducted to develop 3D-printed vaginal delivery systems [17,18,19,20,21,22]. Such attempts have utilized fused disposition modeling or droplet deposition modeling. These techniques represent a layer-by-layer approach of melted polymer and API filament on a build platform. Although this approach is amenable to rapid prototyping and can create precise designs, this could potentially cause the development of rigid parts that may not be comfortable for products that are meant to go inside the body. Nevertheless, these studies demonstrated the ability to generate unique design structures within IVRs to fine-tune drug release rates and promote near complete drug release. However, this approach still involves hot-melt extrusion, which may not be amenable to drugs that are sensitive to heat. 

Here, we fabricated 3D-printed IVRs utilizing continuous liquid interface production (CLIP™) or digital light synthesis (DLS™) to fabricate IVRs in a biocompatible silicone-based resin (SIL30, Carbon Inc., Redwood City, CA, USA) in a fast, translational and layerless process [12,15,23]. CLIP incorporates oxygen into the fabrication process, which prevents polymerization or solidification. Thus, in oxygen dominated areas, such as near the window where oxygen is fed, an area of uncured resin is generated, called the ‘dead zone’, which allows for the continuous and layerless production of smooth and monolithic parts [12,23,24]. Furthermore, although drugs can be incorporated into the resin to generate the drug-loaded IVR, the fabrication process involves heat and UV exposure, which can degrade sensitive APIs. Fortunately, IVR fabrication with CLIP allows for the opportunity to separate ring fabrication and drug incorporation, allowing IVRs to be drug-loaded by absorption mechanisms due to the swelling properties of the silicone poly(urethane) matrix, promoting drug uptake when IVRs are placed in a drug-acetone solution, as previously described [12,16]. 

**Figure 1:**
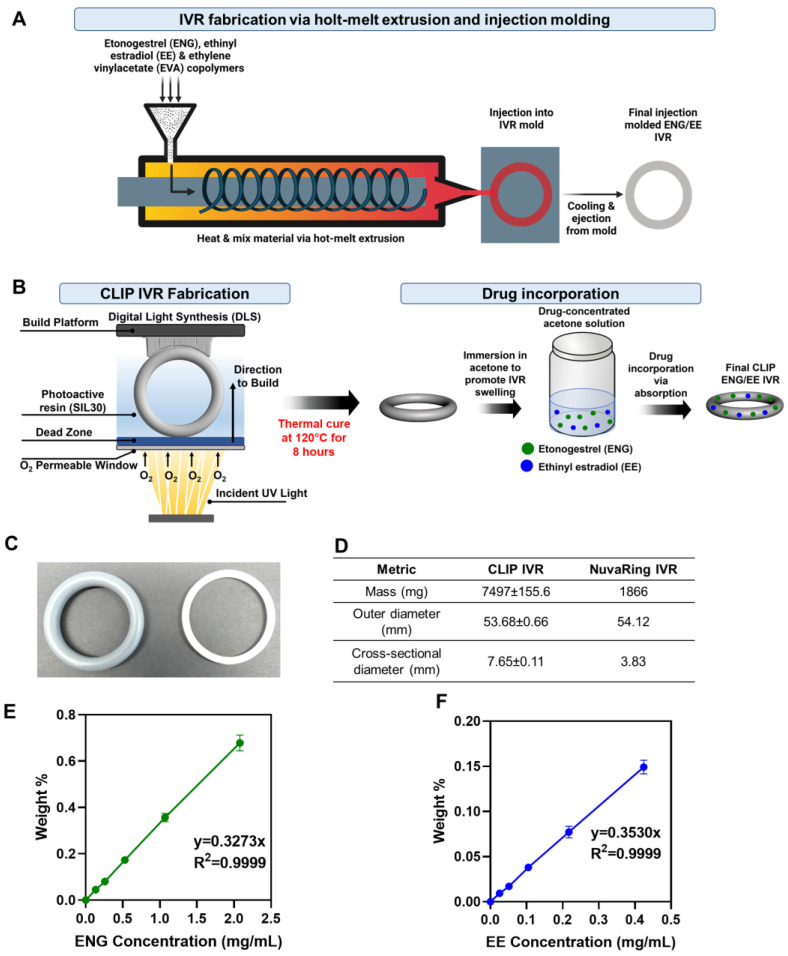
Illustration of IVR manufacturing and loading equations for ENG and EE. (**A**) Schematic of IVR manufacturing with traditional hot-melt extrusion and injection molding. Drugs (ENG and EE) and copolymer (EVA) are poured into the hopper of the injection molding machine. Material is heated and uniformly mixed via hot-melt extrusion and injected into the final IVR mold. Once cooled, the machine ejects the final ENG/EE EVA IVR (NuvaRing). Figure made with BioRender.com. (**B**) IVR manufacturing with CLIP 3D printing (CLIP IVR). Projection of UV light onto photoactive resin (SIL30) promotes polymerization and solidification of the UV-cured IVR. Incorporation of oxygen via an oxygen-permeable window generates a region of uncured resin, known as the ‘dead zone’, to prevent part-attachment to the window. After the UV cure, the IVR undergoes a thermal cure to complete the fabrication process to produce the final product. ENG/EE was incorporated into the IVR via absorption as the IVR swells upon immersion in a drug-containing solution, promoting drug uptake. (**C**) Image of CLIP IVR (**left**) and NuvaRing (**right**). (**D**) Mass and dimensions of CLIP IVR (*n* = 3) and NuvaRing (*n* = 1). (**E**,**F**) Loading equations of ENG and EE in CLIP IVR, respectively, as previously developed and validated [12].

Therefore, we sought to investigate differences in the drug release of common hormonal contraceptives, etonogestrel (ENG) and ethinyl estradiol (EE), from commercially available NuvaRing^®^ and a solid 3D-printed IVR fabricated with CLIP (Figure 1A,B). NuvaRing is a commercially available ethylene vinyl acetate (EVA)-based IVR manufactured by IM for contraception, containing 11.7 mg of ENG and 2.7 mg EE, and is administered every 21 days and removed for 7 days [25]. The 3D-printed IVRs described in this manuscript are fabricated with CLIP (referred to as ‘CLIP IVR’) with a silicone polyurethane-based matrix. ENG and EE were loaded into CLIP IVRs by a solvent-mediated loading process driven by absorption, resulting in similar drug amounts of ENG and EE in the IVR compared to NuvaRing. Although we were able to incorporate the IVR design with CLIP, we fabricated solid CLIP IVRs to gain an understanding of material and drug interactions and to provide head-to-head comparisons to NuvaRing. A head-to-head comparison between NuvaRing and the CLIP IVRs developed in this manuscript will provide an in vitro and in vivo benchmark for delivering ENG and EE vaginally, which will aid in optimizing other vaginal delivery systems delivering ENG and EE. Additionally, this work compares IVR manufacturing techniques and material choices and their impact on ENG and EE release kinetics. Furthermore, to our knowledge, there is no published report on NuvaRing PK in sheep, which is essential information for establishing a preclinical contraceptive benchmark for ENG or EE when administered vaginally in a relevant large animal model. Thus, we sought to determine a preclinical contraceptive benchmark for vaginally delivered ENG and EE in sheep that received NuvaRing to aid in eliminating the need to compare preclinical animal data to available human data for NuvaRing [25]. 

Here, we investigated differences in the in vitro and in vivo release of ENG and EE from CLIP IVRs (3D-printed) and NuvaRing (IM marketed product). First, we assessed the ENG and EE in vitro release from NuvaRing and CLIP IVRs (referred to as ‘CLIP_LOW_ IVRs’) and post-storage in vitro drug release when IVRs were stored in accelerated stability conditions. In these initial studies, CLIP_LOW_ IVRs had a similar ENG/EE loading to NuvaRing. Next, we conducted pharmacokinetic (PK) and safety studies in sheep with NuvaRing and CLIP_LOW_ IVR [26]. Sheep were used as a human model due to their anatomical similarities, such as similar sized vaginal canal and body mass, and are commonly used as a large animal model for assessing PK and the safety of IVRs [27,28,29]. Thus, due to the anatomical similarities between sheep and humans, we were able to utilize the same size IVR and dose to what would be administered in humans. Since there is no published report on NuvaRing PK in sheep, we determined a preclinical contraceptive benchmark for vaginally delivered ENG and EE in sheep that received NuvaRing. 

Leveraging the PK results from the sheep study with NuvaRing, we sought to develop a CLIP_HIGH_ IVR with an increased hormone dose to sustain hormone release for 90 days or longer, with plasma levels greater than or equal to NuvaRing to reduce dosing frequency. This 90-day ENG/EE IVR will be utilized in future studies to develop a long-acting MPT IVR by incorporating an antiretroviral in the IVR for the prevention of HIV and unplanned pregnancy to expand preventative options for women and girls across the globe. 

## 2. Materials and Methods

### 2.1. Materials 

Silicone-urethane-based resin (SIL30) was supplied by Carbon Inc. Etonogestrel (ENG) was purchased from Selleckchem (Houston, TX, USA) (Catalog # S4673). Ethinyl estradiol (EE) was purchased from Sigma Aldrich (Saint Louis, MO, USA) (CAS # 57-63-6). For sheep studies, NuvaRing was purchased through University of Texas Medical Branch’s (UTMB) pharmacy. Sodium acetate, Solutol-HS 15 and high-performance liquid chromatography (HPLC)-grade acetonitrile (ACN) with 0.1% trifluoracetic acid, water with 0.1% trifluoracetic acid, isopropyl alcohol and acetone were purchased from Sigma Aldrich (Saint Louis, MO, USA). 

### 2.2. Computational-Aided Design of 3D-Printed IVRs 

Digital light synthesis (DLS) 3D printing was used to fabricate intravaginal rings, as previously described [12,15,26]. Briefly, a computer-aided design (CAD) file was generated in SolidWorks (Dassault Systèmes, Vélizy-Villacoublay, France, solidworks.com) and converted to an .STL file consisting of unique IVR design parameters. The .STL file was imported into Magics (Materialise, Leuven, Belgium, version 25.0) and a selected unit cell arrayed into the template using the ‘Scaffold’ feature. Rings were exported as an .STL and subsequently imported into Magics again to correct tessellation errors from the file transfer processes. Finally, rings were exported as an .STL for fabrication.

### 2.3. Fabrication of 3D-Printed IVRs with Digital Light Synthesis (DLS)

Solid IVRs were fabricated in SIL30 (a silicone-urethane based resin) with an M1 DLS printer (Carbon, Inc.), as previously described [12,15]. Within the carbon user interface, IVRs were manually positioned vertically on the build platform with supports. The two-part SIL30 resin was thoroughly mixed before dispensing onto the reservoir using a static mixer. Approximately 190 g of SIL30 resin was dispensed onto the M1 reservoir for the vertical printing of 16 human-sized solid IVRs (54 mm outer diameter, 7.6 mm cross-sectional diameter).

### 2.4. Post-Fabrication Treatment for CLIP IVRs

After IVR fabrication via CLIP 3D-printing (CLIP IVRs), CLIP IVRs were cleaned as previously described [12,26]. In brief, IVRs were removed from the build platform and supports were removed. IVRs were subsequently smoothed where the supports were placed to remove any divots or rough edges. IVRs were submerged in isopropyl alcohol while shaking for 1 min and air-dried for 45 min at room temperature. Then, IVRs were placed in an oven at 120 °C for 8 h to facilitate the secondary thermal post-cure, as previously described [12,15,16]. Finally, IVRs were assessed for metrics and dimensions (mass, outer diameter and cross-sectional diameter) and then stored at 4 °C until further use. 

### 2.5. Post-Fabrication Drug Loading and Development of Loading Equations for CLIP IVRs

After IVR fabrication, active pharmaceutical ingredients (APIs) were loaded into the IVR through absorption, resulting in a homogenous distribution of APIs within the SIL30 IVR matrix, as previously described [12,16,26]. In brief, SIL30 parts post-fabrication swell and take up drugs when placed in a drug-containing acetone solution, referred to as a loading solution. Acetone was chosen as the loading solvent due to its ability to swell the SIL30 IVR matrix, highly solubilize ENG (saturation solubility of 89 mg/mL) and EE (saturation solubility of 133 mg/mL), evaporate quickly and be removed easily during the IVR drying process, and is a classified as a Class 3 solvent (low toxicity and low risk to human health) [30]. Here, we developed loading equations, which demonstrate a weight-based loading approach to achieve target drug loadings in our IVRs. Loading equations for ENG and EE were previously developed and validated [12]. In brief, loading equations provide a linear relationship between the drug weight percentage [(mass of drug loaded/mass of IVR) × 100] and the concentration of the drug loading solution. Loading equations were developed by immersing rings in drug-containing acetone solutions (*n* = 3 IVRs per concentration). Drug concentration in the loading solutions was quantified by HPLC analysis. IVRs were incubated in the loading solution at room temperature for 24 h to reach equilibrium swelling, and air-dried overnight followed by drying in an oven under vacuum pressure at 37 °C for 48 h to facilitate acetone removal [12]. To quantify drug loading, dried IVRs were individually placed in acetone for 48 h to facilitate drug extraction. Extraction solutions were quantified by HPLC to determine the amount of drug loaded onto each IVR. Loading equations were determined by graphing the concentration of the loading solutions on the *x*-axis and the weight % on the *y*-axis as previously described and developed [12].

Two iterations of CLIP IVRs were developed within this manuscript: CLIP_LOW_ IVRs (low hormone dose) and CLIP_HIGH_ IVRs (high hormone dose). Both IVRs were generated via CLIP 3D-printing with no change in printing parameters, resin material, or processing times. The only difference between CLIP_LOW_ IVRs and CLIP_HIGH_ IVRs was their amount of ENG and EE loaded in the IVR; specifically, CLIP_HIGH_ IVRs have approximately 3× greater ENG and EE amounts compared to CLIP_LOW_ IVRs. CLIP_LOW_ IVRs that were used for initial in vitro release and sheep studies were loaded in a single step with similar hormone amounts to NuvaRing, approximately 14 mg ENG and approximately 3.5 mg EE for in vitro and in vivo studies, utilizing their respective loading equation. To accomplish this target loading, IVRs of known masses were placed in an acetone solution with ENG and EE (0.58 mg/mL ENG and 0.12 mg/mL EE; 100 mL/IVR) for 24 h to facilitate drug uptake. CLIP_HIGH_ IVRs were loaded with higher hormone concentrations of approximately 40 mg ENG and 10 mg EE, utilizing their respective loading equations. To achieve these higher concentrations, IVRs with known masses were placed in a drug-loaded acetone solution containing 1.6 mg/mL ENG and 0.40 mg/mL EE (100 mL/IVR) for 24 h. All IVRs were removed from drug-concentrated solution and subsequently dried as described above. IVRs were massed daily, and drying was considered complete once the mass of the IVR reached equilibrium. After drying, the IVR (*n* = 1) was extracted in 150 mL of acetone for 48 h to confirm drug loading. The extraction solution was diluted with ACN and quantified by HPLC.

### 2.6. High-Performance Liquid Chromatography (HPLC)

A reverse-phase HPLC analysis was carried out on an Agilent 1260 HPLC system (Agilent Technologies, Santa Clara, CA, USA) with a Diode Array Detector and LC pump with autosampler, as previously described [12]. The stationary phase for analyzing ENG and EE was an Inertsil ODS-3 column (5 μm, 4.6 mm × 150 mm) maintained at 40 °C. Chromatographic separation was achieved by gradient elution with a mobile phase of 95% water and 5% ACN, each including 0.1% trifluoroacetic acid. The flow rate was 1.0 mL/min with a 25 μL injection for a total run time of 25 min. ENG was eluted at 15.2 min and measured at 254 nm (LOD and LOQ of 0.244 µg/mL) and EE was eluted at 13.8 min and measured at 220 nm (LOD of 0.488 µg/mL and LOQ of 0.976 µg/mL).

### 2.7. In Vitro Release Studies

Drug-loaded CLIP_LOW_ IVRs, CLIP_HIGH_ IVRs and NuvaRing were placed in 200 mL of simulated vaginal fluid (SVF) at pH 4 (human vaginal pH [31,32]) or pH 7 (sheep vaginal pH [33]) at 37 °C. The SVF was adapted from Owen and Katz [34], and consisted of 25 mM sodium acetate buffer adjusted to pH 4 (human vaginal pH) or pH 7 (sheep vaginal pH) with hydrochloric acid and 2% Solutol. The addition of Solutol was used to increase the solubility of ENG and EE in the release media and maintain release under sink conditions. Sink conditions were defined as the drug concentration at or below 1/10 of maximum solubility [35] in SVF with 2% Solutol (ENG and EE saturation solubility in SVF with 2% Solutol of 145 μg/mL and 342 μg/mL, respectively). The release media was completely replaced at day 1, day 4 and then weekly to maintain sink conditions. Sample aliquots (1 mL) were collected after 1, 3 and 6 h, daily for a week, weekly for a month and biweekly afterwards. Drug concentration was determined by HPLC analysis with a method developed and validated to analyze ENG and EE in a single run for CLIP_LOW_ IVRs, CLIP_HIGH_ IVRs and NuvaRing. Cumulative drug release was calculated from the HPLC analysis and was normalized to the total mass of drug in the IVR. CLIP_LOW_ IVR and CLIP_HIGH_ IVR in vitro release experiments were conducted in triplicate. NuvaRing in vitro release experiments were conducted with *n* = 1. 

### 2.8. Accelerated Stability Study and Post-Storage In Vitro Release

Drug-loaded CLIP_LOW_ IVRs (*n* = 4) and NuvaRing (*n* = 1) were placed in a glass chamber at 40 °C/75% relative humidity (RH) in a Fisher Scientific Isotemp Incubator (Pittsburgh, PA, USA) [36] for 90 days with previously described methods [12]. A humidity sensor was placed within the glass chamber to confirm 75% RH. A post-storage in vitro release study was conducted in SVF pH 4 to assess differences in drug release after 90 days in accelerated storage conditions compared to baseline (t = 0). CLIP_LOW_ IVR post-storage in vitro release was carried out in triplicate. NuvaRing post-storage in vitro release was conducted with *n* = 1.

### 2.9. Sheep Pharmacokinetics and Safety with ENG/EE CLIP IVR and NuvaRing

Sheep studies were approved by the UTMB Institutional Animal Care and Use Committee, which is PHS/OLAW Assured, USDA registered and AAALAC, International accredited. Female Merino cross-bred sheep were housed indoors in climate-controlled pens with a 12:12 light/dark cycle, fed a pellet and hay diet and had water ad libitum and daily veterinary staff interaction. Sheep were used as a human model due to their anatomical similarities, such as a similar sized vaginal canal and body mass, and are commonly used as a large animal model for assessing PK and the safety of IVRs [27,28,29]. Sheep were enrolled in either a 92-day CLIP_HIGH_ IVR (*n* = 6) study or a 21-day NuvaRing study (*n* = 4), and health checks and weights were obtained prior to examinations. Prior to each study evaluation, the sheep were fasted overnight and then anesthetized with ketamine IM 10 mg/kg and given isoflurane by facemask 1–5% titrated to effect. During the evaluation, colposcopy was performed as previously described and blood, vaginal secretions and vaginal biopsies were obtained [12,28]. 

For the CLIP_HIGH_ IVR studies, colposcopy was performed and samples from blood, vaginal secretions and vaginal biopsies were obtained on study days during the 92 days of IVR use and after removal. Six sheep were enrolled in the study. One sheep (sheep ID 1190) had an expulsion of the IVR on day 12; however, it was retrieved and replaced on day 14, and she continued in the study without another expulsion. IVRs were replaced so that 3 sheep (sheep IDs 1172, 1107 and 1100) completed the full 92 days with the use of a single IVR and 3 sheep (sheep IDs 1190, 1045 and 1063) completed 60 days using a single IVR, followed by a 4-day washout, and were then administered a different IVR for 28–30 days (*n* = 2, sheep IDs 1045 and 1190). Used IVRs were removed, stored at −80 °C and later tested for residual drug. 

For the 21-day NuvaRing study, colposcopy was performed and samples from blood, vaginal secretions and vaginal tissue were obtained at baseline, day 1, 3, 7, 14 and 21 in 4 sheep. The IVRs were removed at day 21 to simulate human use of the product and samples (plasma, vaginal secretions and vaginal biopsies) were collected 7 days after removal. 

Biopsies were evaluated for PK and histological analysis (H&E) for sheep that were administered NuvaRing and CLIP_HIGH_ IVRs. Biopsies for H&E were processed similarly, as previously described [12]. In brief, vaginal biopsies were collected and immediately immersed in 10% Neutral Buffered Formalin and allowed to be fixed for 72 h prior to routine processing to paraffin, sectioning to 5 µm and hematoxylin and eosin staining [12]. Microscopic evaluation of the samples was performed by a Board-certified veterinary pathologist and microscopic findings were graded on a scale of 0–5 (0 = no findings; 1 = minimal findings; 2 = mild findings; 3 = moderate findings; 4 = marked findings; 5 = severe findings). 

It is important to note that sheep PK and safety studies with CLIP_LOW_ IVRs were previously performed and described in another manuscript [12] and were used for comparing ENG/EE PK to sheep that received NuvaRing and the CLIP_HIGH_ IVRs. Furthermore, sheep studies with CLIP_LOW_ IVRs were co-formulated with an antiretroviral, but hormones release kinetics from the IVRs were similar with or without the addition of the antiretroviral [12].

### 2.10. Analytical Pharmacokinetic Analysis

Plasma was separated from EDTA-treated whole blood by centrifugation. EE and ENG in sheep plasma were extracted by liquid–liquid extraction with their isotopically labeled internal standards, EE-d4 and ENG-d7. Analytes were separated using reverse-phase chromatography via a Waters Atlantis T3 (50 mm × 2.1 mm, 3.0 µm particle size) analytical column for EE or a Phenomenex Synergi Fusion-RP (50 mm × 2.0 mm, 4.0 µm particle size) for ENG. An AB Sciex API-6500 triple quadruple mass spectrometer was used to detect analytes and internal standards under positive (ENG, ENG-d7,) or negative (EE, EE-d4) ion electrospray conditions. The precision and accuracy of the calibration standards and quality control samples were within 30% (EE) or 15% (ENG) with the following lower limits of quantitation (LLOQs): 0.005 ng/mL (EE) and 0.100 ng/mL (ENG).

Mucosal fluid collected onto Merocel sponges was eluted by adding 2 mL of 70:30 acetonitrile/water to 15 mL Falcon tubes containing a single sponge, vortexing for 1–2 min and aliquoting into a 1.5 mL microcentrifuge tube. Weighed tissues were transferred into Precellys^®^ hard tissue reinforced metal bead kit tubes (Cayman Chemical Company, Ann Arbor, MI, USA) containing 0.500 mL of 70:30 acetonitrile/water, homogenized and then centrifuged. Following protein precipitation and liquid–liquid extraction for the fluid and tissue samples with their respective isotopically labeled internal standards, EE and ENG were separated using reverse-phase chromatography via an Agilent Pursuit 3 Diphenyl (50 × 2.0 mm, 3.0 µm particle size) analytical column. An AB Sciex API-6500 triple quadruple mass spectrometer was used to detect analytes and internal standards under positive (ENG, ENG-d7) or negative (EE, EE-d4) ion electrospray conditions. The precision and accuracy of the calibration standards and quality control samples were within 20% for the following LLOQs: 0.025 ng/mL (EE) and 0.050 ng/mL (ENG). Tissue sample final concentrations were normalized to the tissue mass of the collected tissue sample and reported in ng/g units.

### 2.11. CLIP_HIGH_ IVR and NuvaRing Ex Vivo Residual Drug Quantification

To determine the residual drug amount in IVRs that were retrieved after completed sheep studies, CLIP_HIGH_ IVRs were placed in 150 mL of acetone and NuvaRings were placed in 100 mL of chloroform for 48 h to facilitate extraction. The extraction solution was diluted with ACN and quantified with HPLC.

### 2.12. Statistical Analysis

Statistical analyses were performed in GraphPad Prism 9 (GraphPad Software, Inc., La Jolla, CA, USA). Two-way ANOVA tests were performed with respect to each in vitro release sampling timepoint and IVR technology (CLIP IVR_LOW_ or NuvaRing) for the entire study duration. A Sidak’s multiple comparisons test was then performed to analyze differences in (1) cumulative in vitro ENG and EE release from CLIP_LOW_ IVR and NuvaRing at each sampling timepoint for the entire study duration, (2) post-storage cumulative in vitro ENG and EE release compared to release at baseline at each sampling timepoint for the entire study duration and (3) drug concentration in sheep plasma, vaginal tissue and vaginal fluids after the administration of CLIP IVRs or NuvaRing at each sampling timepoint for the entire study duration. For all statistical tests, a *p* value of <0.05 was considered significant (95% confidence level). 

## 3. Results and Discussion

### 3.1. IVR Fabrication with Continuous Liquid Interface Production (CLIP™) 

CLIP IVRs were created using computer-aided design (CAD) and fabricated with CLIP 3D printing using a two-part silicone-urethane (SIL30, Carbon Inc) resin. Upon printing, the dual resin selectively solidifies by free radical polymerization mechanisms initiated by exposure to ultraviolet (UV) light [12,15,16,23] (Figure 1B). Additionally, oxygen was fed into the system and is a known inhibitor of polymerization [12,15,16,23,24] (Figure 1B). Thus, a region of uncured resin is generated in an oxygen dominated areas, specifically near the window through which oxygen is fed, referred to as the ‘dead zone’ [15,16,23,24]. Ultimately, the presence of the dead zone prevents the solidified part from attaching to the window, resulting in the continuous and layerless fabrication of the IVR. After the UV cure, IVRs are exposed to an 8 h thermal post-cure to produce the final IVR [15]. NuvaRing (manufactured by IM) was procured by a pharmacy and used in the studies described in this manuscript. 

Mechanical properties of CLIP IVRs and NuvaRing were evaluated by compression testing, as previously described [15]. In brief, CLIP IVRs and NuvaRing (EVA IVR) exhibited a load at 50% compression of approximately 1.8 N and 2.6 N, respectively [15]. Differences in compressive forces between the CLIP IVR and NuvaRing are likely due to differences in the material and IVR dimensions. Figure 1C,D demonstrate the final CLIP IVR with their respective mass and dimensions compared to NuvaRing. CLIP IVR and NuvaRing share a similar outer diameter and CLIP IVR elicits a cross-sectional diameter and mass about 2-fold and 4-fold greater than NuvaRing, respectively (Figure 1D). 

### 3.2. Post-Fabrication Drug Loading and Loading Equations

Unlike IVR traditional manufacturing by hot-melt extrusion and IM, IVRs generated with CLIP allow for the separation of ring fabrication and drug incorporation. This is of great importance, as products produced by IM are generated under high heat and pressures, which could limit material or API choices. Although this is not an issue with NuvaRing, as EVA and ENG/EE can withstand these harsh conditions, the inherent high heat and pressure could be a limitation when considering more sensitive materials or APIs. 

ENG and EE were incorporated into the CLIP IVR in a single step via a solvent-mediated post-loading process [16], as previously described [12]. In brief, CLIP IVRs swell considerably when placed in a drug–acetone solution due to hydrophobic interactions between the silicone material (SIL30) and the solvent, eliciting absorption and complete intercalation of APIs within the CLIP IVR matrix [16]. We previously developed a weight-based loading approach to achieve target drug loading in our IVRs, referred to as loading equations, as previously described [12]. Loading equations for ENG and EE that were previously developed and validated [12] are shown in Figure 1E and F, respectively. Two iterations of CLIP IVRs were developed in this manuscript: CLIP_LOW_ IVRs (low hormone dose) and CLIP_HIGH_ IVRs (high hormone dose). For initial studies, CLIP_LOW_ IVRs contained approximately 14 mg ENG, and approximately 3.5 mg EE. To sustain ENG/EE release for at least 90 days in vivo, we developed CLIP_HIGH_ IVRs in which the hormone content was increased to 40 mg ENG and 10 mg EE. 

### 3.3. In Vitro Release and Post-Storage Release Studies

**Figure 2:**
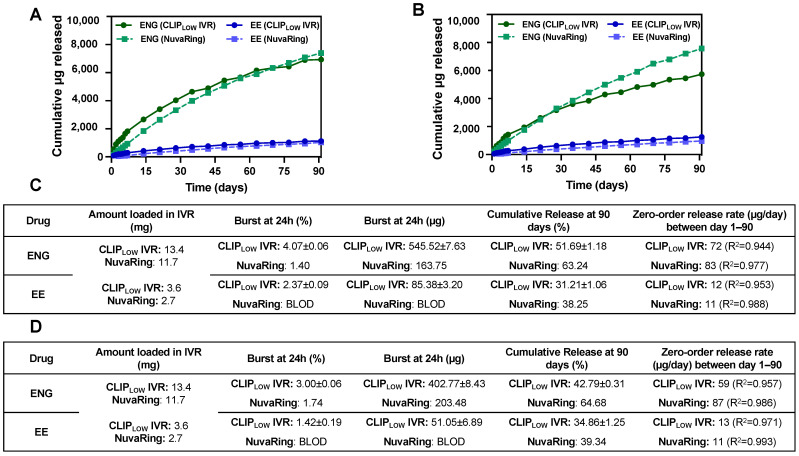
In vitro release of ENG/EE from CLIP_LOW_ IVR and NuvaRing. (**A**) Cumulative μg in vitro release of ENG/EE from CLIP_LOW_ IVR and NuvaRing. (**B**) Cumulative μg in vitro release of ENG/EE from CLIP_LOW_ IVR and NuvaRing after 90 days of storage under accelerated stability conditions (40 °C/75% relative humidity). (**C**,**D**) Summary of release kinetics of in vitro release of ENG/EE from CLIP_LOW_ IVR and NuvaRing at baseline and after 90 days of storage under accelerated stability conditions, respectively. BLOD represents ‘below limit of detection’ from HPLC analysis. CLIP_LOW_ IVR release studies were performed in triplicate (*n* = 3) and NuvaRing in vitro release studies were performed with *n* = 1. All release studies were performed in SVF + 2% Solutol at pH 4 release media at 37 °C. The maximum standard deviation for in vitro cumulative release was less than 2%.

We assessed the in vitro release of ENG/EE from CLIP_LOW_ IVRs and commercially available NuvaRing in SVF pH 4 (human vaginal pH) for 90 days. CLIP_LOW_ IVRs elicited slightly higher hormone loading compared to NuvaRing to account for a potentially higher burst release. Specifically, CLIP_LOW_ IVRs were loaded with approximately 13.4 mg ENG and 3.6 mg EE compared to 11.7 mg ENG and 2.7 mg EE in commercially available NuvaRing [25]. CLIP_LOW_ IVRs elicited a greater burst release of both hormones compared to NuvaRing (Figure 2A,C). This is likely attributed to the fact that CLIP_LOW_ IVRs represent a matrix-type delivery system where the drug is homogenously distributed throughout the IVR, and thus drug particles closer to the surface of the IVR release faster when exposed to the release media, resulting in increased burst release within the first 24 h. Conversely, NuvaRing is a reservoir-type delivery system in which drugs are dispersed in the IVR core and surrounded by a drug-free polymeric rate-controlling membrane, which limits the burst release [37]. 

The ENG release from CLIP_LOW_ IVR was slightly greater compared to NuvaRing and statistically different (*p* < 0.05) for the first 49 days, as shown in Figure 2A. After 49 days, the release of ENG from NuvaRing (83 µg/day) was slightly greater compared to CLIP_LOW_ IVR (72 µg/day). The release of EE from CLIP_LOW_ IVR was greater (12 µg/day) and statistically different (*p* < 0.05) compared to its release from NuvaRing (11 µg/day) throughout the entire 90-day release duration.

Assessing drug release after storage is essential to determining IVR shelf life and storage conditions. CLIP_LOW_ IVR and NuvaRing were stored in an accelerated stability chamber under 40 °C and 75% relative humidity (RH) for 90 days. After 90 days in storage, the CLIP_LOW_ IVR elicited significantly slower (*p* < 0.05) ENG release and comparable EE release when compared to baseline (Figure 2B,D). The post-storage EE release from CLIP_LOW_ IVR was not significantly different compared to the baseline. Drug release from CLIP_LOW_ IVR after storage in accelerated stability conditions was quite different from the baseline, particularly for ENG, likely due to the high storage temperature, promoting drug and material interactions and causing changes in drug release kinetics. It was previously reported that levonorgestrel, a progestin with similar chemical functional groups and structure to ENG, covalently bonds to silicone elastomers in IVR matrices due to hydrosilylation reactions [38,39]. This irreversible chemical bonding of the progestin and silicone elastomer can result in an inability of the drug to be released from the IVR and can be further influenced when exposed to heat [38,39]. Thus, we hypothesize that a similar phenomenon may be occurring with ENG in our silicone-based CLIP_LOW_ IVR, potentially explaining the considerably slower ENG release after storage in accelerated stability conditions. 

Conversely, NuvaRing elicited similar ENG and EE release after 90 days in storage when compared to the baseline (Figure 2B,D). Overall, NuvaRing elicited a greater release of ENG and lower EE release compared to CLIP_LOW_ IVR after 90 days in storage. Due to the considerable changes in drug release post-storage for the CLIP_LOW_ IVR, in the future we plan to perform release studies post-storage in 4 °C, as that is the established storage condition for the commercially available NuvaRing [25]. 

### 3.4. Sheep Pharmacokinetics Studies 

A pilot PK sheep study was performed with CLIP_LOW_ IVR and NuvaRing to assess differences in ENG/EE release in vivo. To account for an expected greater burst release in vivo, we increased the ENG/EE loading in our CLIP IVR to approximately 14 mg ENG and 3.5 mg EE. Additionally, it is important to note that PK data from a CLIP MPT IVR that contained an equivalent dose of EE/ENG to CLIP_LOW_ IVR, in combination with an antiretroviral drug, have previously been described in another manuscript [12]. However, we previously demonstrated no impact on in vitro hormone release when combined with the antiretroviral [12], which we further investigated in the in vivo sheep studies with EE/ENG CLIP IVRs. 

**Figure 3:**
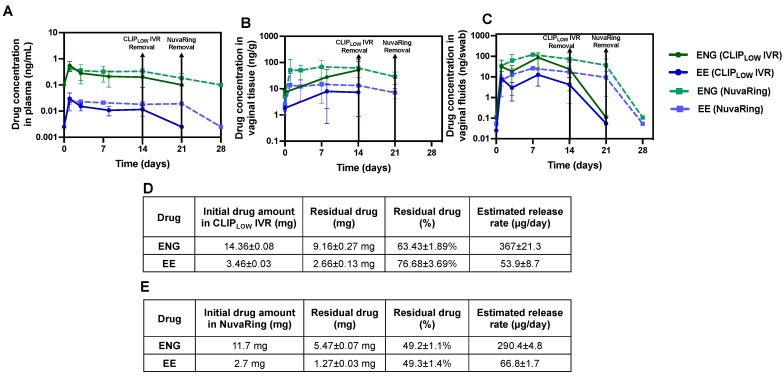
Pharmacokinetics of CLIP_LOW_ IVR and NuvaRing in sheep. ENG/EE levels in sheep (average ± standard deviation) from CLIP_LOW_ IVR (*n* = 4) and NuvaRing (*n* = 4) in (**A**) plasma, (**B**) vaginal tissue and (**C**) vaginal fluids. LLOQ of ENG in plasma, vaginal tissue and vaginal fluid is 0.2 ng/mL, 17 ng/g and 0.215 ng/swab, respectively. LLOQ of EE in plasma, vaginal tissue and vaginal fluid is 0.005 ng/mL, 4.2 ng/g and 0.108 ng/swab, respectively. Samples that were below the limit of quantification were represented as LLOQ/2. (**D**,**E**) Summary table of residual drug quantification and estimated in vivo release rates after CLIP_LOW_ IVR and NuvaRing removal, respectively. Individual replicates are shown in Figure S1.

As previously described [12], CLIP_LOW_ IVRs (0.25 mg/kg ENG and 0.055 mg/kg EE) were inserted into four female sheep and the plasma, vaginal tissue and vaginal fluids were longitudinally collected for 14 days. CLIP_LOW_ IVRs were removed after 14 days and plasma, vaginal tissue and proximal vaginal fluids were collected at 7 days post-IVR-removal to assess drug elimination and the reversibility of the contraception. Commercially available NuvaRings (0.17 mg/kg ENG and 0.039 mg/kg EE) were inserted into four female sheep for 21 days to mimic the clinical use of this product, and plasma, vaginal tissue and vaginal fluids were collected. NuvaRings were removed after 21 days, and plasma and vaginal fluids samples were collected at 7 days post-removal. Figure 3A–C demonstrates the PK of ENG/EE from CLIP_LOW_ IVR compared to the NuvaRings for plasma, vaginal tissue and vaginal fluid. In all matrices, NuvaRing elicited higher levels of both ENG and EE compared to CLIP_LOW_ IVR. Specifically, NuvaRing elicited drug levels that were up to 1.7-fold higher for ENG and up to 2.0-fold higher for EE in plasma. In vaginal tissue, NuvaRing resulted in up to 2.5-fold higher ENG and up to 1.9-fold higher EE compared to CLIP_LOW_ IVRs, and similarly vaginal fluid levels were up to 3.4-fold higher for ENG and up to 4.4-fold higher for EE with NuvaRing compared to CLIP_LOW_ IVR. The slightly higher hormone concentrations in sheep that received NuvaRing may be attributed to differences in IVR material (EVA in NuvaRing and silicone-polyurethane in CLIP_LOW_ IVR) as material–drug interactions and drug solubility in the material are known to impact drug release kinetics [40,41]. Additionally, the NuvaRing elicits a smaller cross-sectional diameter (4.0 mm cross-sectional diameter) compared to CLIP_LOW_ IVR (7.6 mm cross-sectional diameter), which may increase drug release due to smaller drug diffusion distances [16]. However, despite the higher concentrations of ENG and EE in all matrices from NuvaRing compared to CLIP_LOW_ IVR, the observed levels were not statistically significantly different (*p* > 0.05). 

After CLIP_LOW_ IVR and NuvaRing were removed from the sheep, ENG and EE levels fell below the lower limit of quantification within 7 days (Figure 3A–C), demonstrating a rapid clearance of the hormones. Furthermore, after the 14-day study, CLIP_LOW_ IVRs had approximately 63% residual ENG and 77% residual EE (Figure 3D). After the 21-day study, NuvaRing had approximately 49% of both ENG and EE remaining (Figure 3E). In vivo release rates were estimated (initial drug amount—residual drug amount divided by study duration) and showed that CLIP_LOW_ IVR achieved release rates of 367 µg ENG/day and 54 µg EE/day and NuvaRing achieved release rates of 290 µg ENG/day and 67 µg EE/day (Figure 3D,E). However, it is important to note that we cannot make a direct comparison of residual drug amounts and estimated in vivo release rates between CLIP_LOW_ IVR and NuvaRing since the study durations were different (14 days for CLIP_LOW_ IVR and 21 days for NuvaRing). 

Ultimately, the results from this study determined a preclinical benchmark for vaginally delivered ENG and EE in sheep based on average trough concentrations from the commercially available NuvaRing (average plasma concentrations at day 21 of 0.182 ng ENG/mL and 0.0192 ng EE/mL). These results also demonstrated that a higher dose of ENG and EE in the CLIP_LOW_ IVR is required to achieve drug concentrations greater than or equal to NuvaRing over the target duration of 90 days. 

### 3.5. In Vitro Release and Sheep Pharmacokinetics of ENG/EE from CLIP_HIGH_ IVR with Increased Hormone Dose 

**Figure 4:**
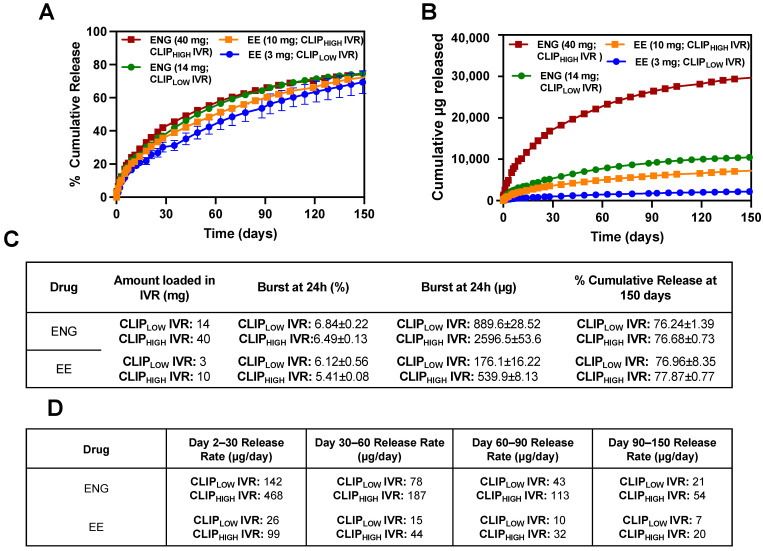
In vitro release of ENG/EE from CLIP_HIGH_ IVR compared to CLIP_LOW_ IVR. (**A**) Cumulative (%) in vitro release of ENG/EE from CLIP_HIGH_ IVR compared to CLIP_LOW_ IVR. (**B**) Cumulative (µg) in vitro release of ENG/EE from CLIP_HIGH_ IVR compared to CLIP_LOW_ IVR. (**C**) Summary table of in vitro release profiles. (**D**) Summary table of in vitro release rates. All studies were carried out in triplicate in SVF pH 7 (sheep vaginal pH) release media. Each release curve represents the average ± standard deviation. Maximum standard deviation for in vitro cumulative release is less than 2%.

Leveraging the results from the sheep studies conducted with CLIP_LOW_ IVR and NuvaRing, we sought to increase ENG/EE drug loading in the IVR to develop the CLIP_HIGH_ IVR to sustain drug release in vivo for ≥90 days while achieving drug concentrations in plasma greater than or equal to NuvaRing levels in sheep. This optimized CLIP_HIGH_ IVR with ENG/EE will be used to develop a long-acting (≥90 days) MPT IVR with an antiretroviral in future studies. Thus, we increased the drug loading of ENG/EE within the CLIP_LOW_ IVR by approximately 3-fold to 40 mg ENG and 10 mg EE. Figure 4 demonstrates the in vitro release of the high hormone dose (CLIP_HIGH_ IVR; 40 mg ENG and 10 mg EE) compared to the original hormone dose (CLIP_LOW_ IVR; 14 mg ENG and 3 mg EE) for 150 days. The release studies were performed in SVF pH 7 (sheep vaginal pH) to best represent the sheep vaginal environment. Notably, the ENG/EE in vitro release from the IVRs was sustained for 150 days, with a low burst release (<10% burst within 24 h), and elicited proportionality between the loading dose and the release rate. In other words, when the loading dose increased by approximately 3-fold, the release rate also increased by approximately 3-fold.

**Figure 5:**
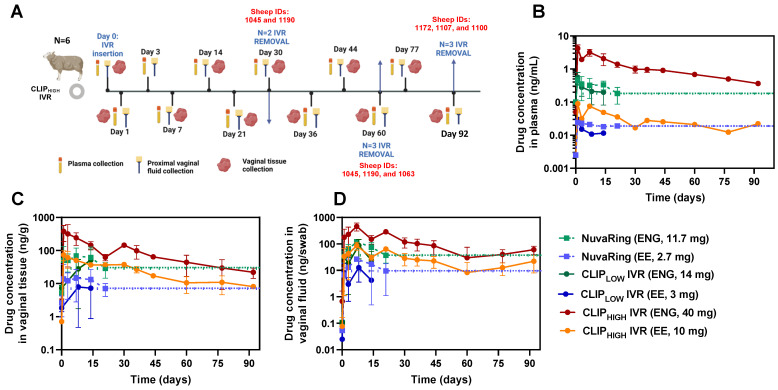
Sheep pharmacokinetics of ENG/EE after 92 days of CLIP_HIGH_ IVR administration. (**A**) Study design of a 92-day PK study with CLIP_HIGH_ IVR in female sheep. ENG/EE concentrations in sheep (*n* = 3 sheep that underwent the entire 92-day study) in (**B**) plasma, (**C**) vaginal tissue and (**D**) vaginal fluids from CLIP_LOW_ IVR, CLIP_HIGH_ IVR and NuvaRing. Each curve represents the average ± standard deviation. Dashed lines indicated the last concentration (day 21) of ENG (dashed green line) and (dashed blue line) from the NuvaRing sheep study extended to 92 days for comparative purposes only. Individual replicates for sheep that underwent the 92-day study duration with CLIP_HIGH_ IVR are shown in Figure S2. Individual replicates for CLIP_LOW_ IVR and NuvaRing are shown in Figure S1.

Based on the promising in vitro release data, we conducted a PK study with the CLIP_HIGH_ IVRs in six (*n* = 6) female sheep to determine whether systemic ENG/EE levels greater than or equal to NuvaRing can be achieved for 90 days or longer. The study design for the sheep PK study is illustrated in Figure 5A. Plasma, vaginal tissue and vaginal fluid were collected for up to 92 days. At days 30, 60 and 92, CLIP_HIGH_ IVRs were removed from sheep to assess the elimination of hormones after IVR termination and to quantify the residual drug amount in the IVR after removal. Specifically, *n* = 3 sheep (sheep IDs 1172, 1107 and 1100) were studied for 92 days, followed by a 3-day washout. An additional *n* = 3 sheep (sheep IDs 1190, 1045 and 1063) were studied for 60 days, followed by a 4-day washout. After the 4-day washout, *n* = 2 sheep (sheep ID 1045 and 1190) were re-used and administered new CLIP_HIGH_ IVRs and studied for 30 days followed by a 3-day washout period. Figure 5B–D shows the ENG/EE levels in plasma, vaginal tissue and vaginal fluids for sheep (*n* = 3) that were subject to the entire 92-day study duration. ENG and EE levels were compared to sheep that received NuvaRing (11.7 mg ENG and 2.7 mg EE) and sheep that received CLIP_LOW_ IVRs (14 mg ENG and 3 mg EE) in prior studies described in this manuscript. 

Notably, after 92 days of use, CLIP_HIGH_ IVRs achieved levels that were greater than or equal to levels obtained with NuvaRing in all matrices. Specifically, at day 92, CLIP_HIGH_ IVR elicited drug levels that were approximately 2.0-fold higher for ENG and 1.2-fold higher for EE in plasma and exhibited similar vaginal tissue and fluid levels for ENG and EE compared to day 21 ENG/EE levels from NuvaRing. At earlier timepoints, such as between days 1 and 21, ENG and EE levels from the CLIP_HIGH_ IVR were 4.9–8.0-fold higher for ENG and 1.7–3.2-fold higher for EE in plasma; 1.8–31.6-fold higher for ENG and 2.1–39.5-fold higher for EE in vaginal tissue; and 2.7–11.9-fold higher for ENG and 2.0–8.7-fold higher for EE in vaginal fluids compared to NuvaRing. These considerably higher levels of ENG and EE at early timepoints are not necessary for contraception and can potentially cause uncomfortable or unwanted side effects [42,43], and thus future studies will include the further optimization of the hormone dose to reduce drug concentrations while still achieving our 90-day duration target. 

Furthermore, between days 1 and 14, CLIP_HIGH_ IVR resulted in average plasma concentrations (1.6 ng/mL ENG and 0.036 ng/mL EE at day 14) that were 6.8–12.3-fold higher for ENG and 2.9–7.0-fold high for EE compared to CLIP_LOW_ IVRs. In vaginal tissue (average concentration of 104.5 ng/mL ENG and average concentration of 30.8 ng/mL EE at day 14), levels were 3.2–8.8-fold higher and 3.0–7.4-fold higher for ENG and EE, respectively, compared to CLIP_LOW_ IVRs. Similarly, ENG and EE concentrations in vaginal fluids were 3.7–7.9-fold higher for ENG (average concentration at day 14 of 140.7 ng/mL) and 4.4–10.5-fold higher for EE (average concentration at day 14 of 23.9 ng/mL) compared to levels obtained with the CLIP_LOW_ IVRs. Although we saw exact dose proportionality in vitro between the CLIP_HIGH_ IVR and CLIP_LOW_ IVR, this was not directly translated in vivo, where we observed a greater than a 3-fold increase in hormone levels in all matrices despite the 3-fold increase in ENG and EE dose. 

**Figure 6:**
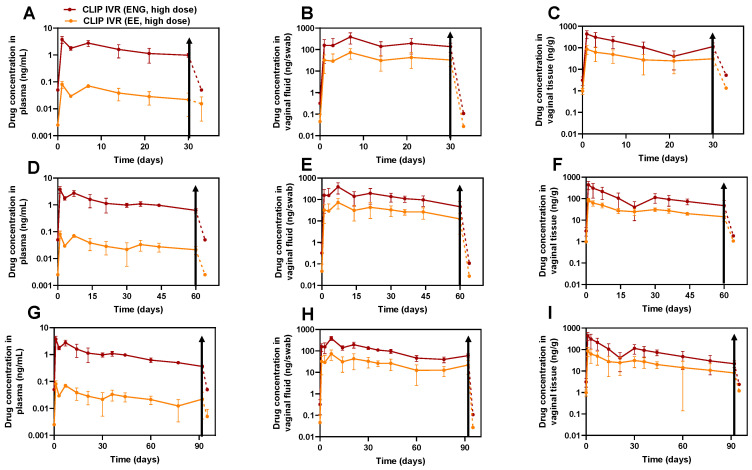
Assessment of PK and PK tail after CLIP_HIGH_ IVR removal. (**A**–**C**) CLIP_HIGH_ IVR removal at 30 days post-IVR administration in (**A**) plasma, (**B**) vaginal fluid and (**C**) vaginal tissue. (**D**–**F**) CLIP_HIGH_ IVR removal at 60 days post-IVR administration in (**D**) plasma, (**E**) vaginal fluid and (**F**) vaginal tissue. (**G**–**I**) CLIP_HIGH_ IVR removal at 92 days post-IVR administration in (**G**) plasma, (**H**) vaginal tissue and (**I**) vaginal fluid. Drug concentration curves are plotted as average ± standard deviation. Data from all sheep (*n* = 6) were pooled together for timepoints when IVRs were inserted. At day 30 post-IVR administration, *n* = 2 IVRs were removed from sheep (**A**–**C**) and at days 60 and 92 post-IVR administration, *n* = 3 IVRs were removed (**D**–**I**). Solid lines indicate when IVRs were inserted, and dashed lines indicate post-IVR removal. Black arrows indicate the time at which IVR was removed. LLOQs of ENG in plasma, vaginal tissue and vaginal fluid were 0.1 ng/mL, 5.11 ng/g and 0.215 ng/swab, respectively. LLOQs of EE in plasma, vaginal tissue and vaginal fluid were 0.005 ng/mL, 2.55 ng/g and 0.054 ng/swab, respectively. Samples that were below the limit of quantification were represented as LLOQ/2. Individual replicates are shown in Figure S3.

Additionally, we assessed contraceptive reversibility by removing CLIP_HIGH_ IVRs at days 30, 60 and 92 post-administration and collecting plasma, vaginal tissue and vaginal fluid 3 days post-IVR removal for sheep in the 30-day and 92-day cohort and 4 days post-IVR-removal for sheep in the 60-day cohort. Notably, across all matrices, the removal of CLIP_HIGH_ IVRs at days 30 (Figure 6A–C), 60 (Figure 6D–F) and 92 (Figure 6G–I) resulted in a rapid elimination of ENG and EE, with levels falling below the drug’s lower limit of quantification within 3 or 4 days post-IVR removal, demonstrating fast contraceptive reversibility despite an increased hormone dose. Specifically, we observed an up to 10-fold decline in drug concentrations in plasma, an up to 1000-fold decline in drug concentrations in vaginal fluid, and an up to 100-fold decline in drug concentrations in vaginal tissues within 4 days post-IVR removal. 

**Figure 7:**
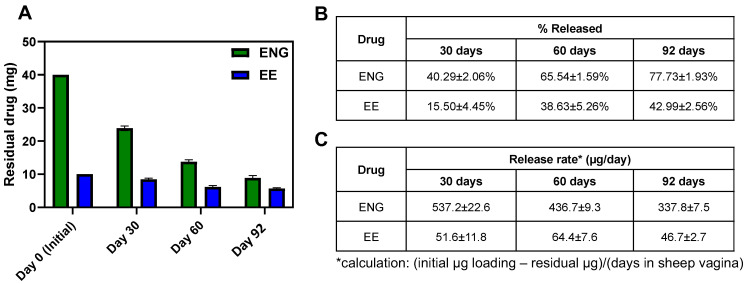
Quantification of residual drug and estimation of in vivo release rates. (**A**) Residual drug ENG/EE after CLIP_HIGH_ IVR removal at days 30, 60 and 92 post-administration. (**B**) % ENG/EE released in vivo after IVR removal. (**C**) Estimation of ENG and EE in vivo release rates based on residual drug content after IVR removal. Data represent average ± standard deviation for *n* = 2 sheep for day 30 and *n* = 3 sheep for days 60 and 92.

Furthermore, residual drug content was quantified for CLIP_HIGH_ IVRs after ring removal at 30, 60 and 92 days post-administration. (Figure 7A,B). After 92 days of use, there was approximately 23% and 52% of ENG and EE remaining, respectively, demonstrating potential for the IVR to release hormones for longer than 92 days. In addition, estimated release rates were calculated based on residual drug content in the CLIP_HIGH_ IVR (initial drug amount—residual drug amount divided by study duration) and ranged between 337 and 537 µg ENG/day and 46 and 52 µg EE/day throughout the 92-day study duration (Figure 7C). 

Ultimately, these data suggest that the CLIP_HIGH_ IVR elicited drug levels that were equivalent to or higher than NuvaRing and had the potential to release for more than 90 days. Building on these promising results, future studies will investigate the formulation of an antiretroviral in the CLIP_HIGH_ IVR for the development of a long-acting (≥90 day) MPT IVR for the prevention of HIV and unintended pregnancy. 

### 3.6. CLIP_HIGH_ IVR and NuvaRing Sheep Safety Studies

Sheep from the CLIP_HIGH_ IVR and the NuvaRing studies were evaluated for safety by general health evaluations, colposcopy and histopathological analysis (H&E). We previously reported on the safety of CLIP_LOW_ IVRs when co-formulated with an antiretroviral, and they were considered safe and showed no signs of chronic toxicity [12]. Sheep from the CLIP_HIGH_ IVR and the NuvaRing studies had general health evaluations conducted by veterinary staff during the studies, as well as colposcopy evaluation of the vaginal mucosa by the study team to determine the safety of the IVRs. Sheep were healthy and showed no signs of distress, lack of appetite, or weight loss during the studies. With the use of the colposcopy, there were no signs of toxicity such as a disruption of the mucosa, bleeding or ulceration. Findings that have been historically seen at baseline or during the use of placebo such as mild to moderate erythema and petechiae were seen throughout the studies. Additionally, diffuse vasculature was also seen, which was likely related to the effects of the local administration of ENG and EE and not an indication of toxicity.

**Figure 8:**
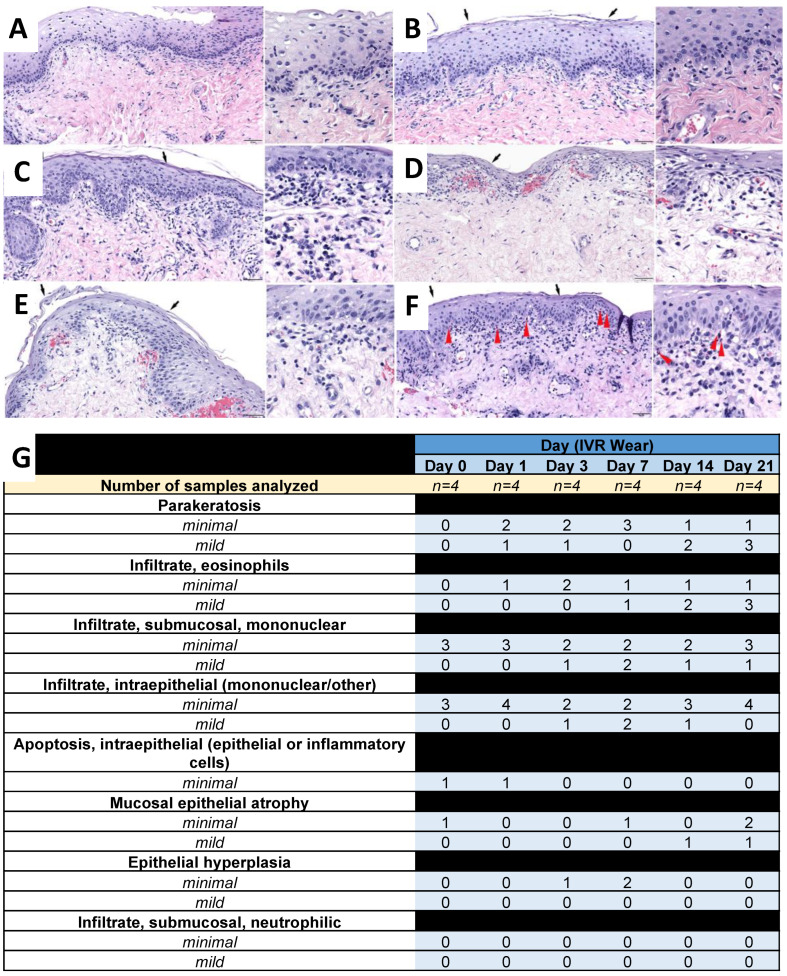
Sheep safety analysis for NuvaRing. Histological images of vaginal biopsies from sheep administered NuvaRing (with higher magnification insert) at (**A**) day 0, (**B**) day 1, (**C**) day 3, (**D**) day 7, (**E**) day 14 and (**F**) day 21 post-IVR insertion. Red arrowheads denote examples of eosinophils. Black arrows denote parakeratosis. Scale bar = 50 µm. (**G**) Histological scoring summary based on H&E images. Individual scores from each sheep are presented in Figure S4. Day 0 represents the baseline with no IVR use.

**Figure 9:**
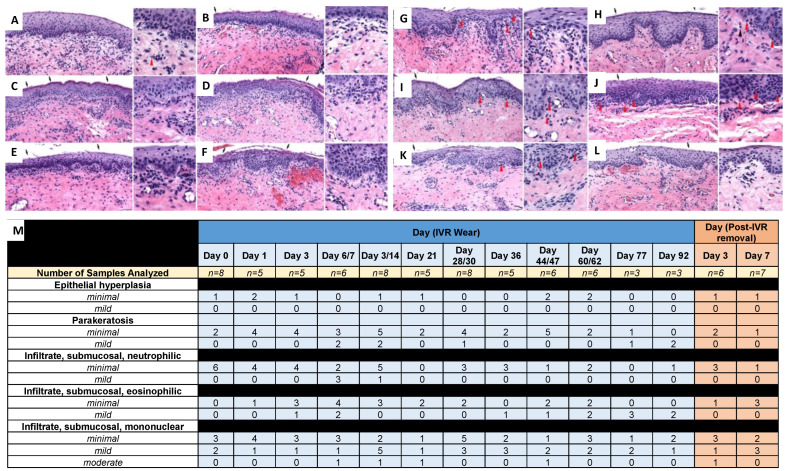
Sheep safety analysis for CLIP_HIGH_ IVR. Histological images of vaginal biopsies from sheep administered CLIP_HIGH_ IVR (with higher magnification insert) at (**A**) day 0, (**B**) day 1, (**C**) day 3, (**D**) day 7, (**E**) day 14, (**F**) day 21, (**G**) day 30, (**H**) day 60, (**I**) day 77 and (**J**) day 92, and (**K**) day 3 post-IVR removal and (**L**) day 7 post-IVR removal. Red arrowheads denote examples of eosinophils. Black arrows denote parakeratosis. Scale bar = 50 µm. (**M**) Histological scoring summary based on H&E images for 92 days. Individual scores from each sheep are presented in Figures S5–S7. Day 0 represents the baseline with no IVR use.

Vaginal biopsies were collected and stained with H&E for histological analysis (Figure 8 and Figure 9). There were no adverse IVR-related findings in the vaginal biopsies evaluated in sheep that received NuvaRing (Figure 8) or CLIP_HIGH_ IVRs (Figure 9). IVR-related findings were mechanistic in nature and attributable to estrogen stimulation. These findings included increased vaginal epithelial parakeratosis and submucosal/intraepithelial infiltrates of eosinophils; these findings were still present at days 3 and 7 post-ring removal in sheep that received the CLIP_HIGH_ IVR. Additionally, sheep that received the CLIP_HIGH_ IVR and NuvaRing elicited an increased incidence of minimal parakeratosis that was evident on days 1 and 3 post-ring insertion; an increased incidence and severity of parakeratosis (up to mild) was evident at and after day 6 or 7 post-ring insertion. The variability of parakeratosis between timepoints likely reflects the biopsy sampling location. Submucosal and/or intraepithelial infiltrates of eosinophils were not evident in Day 0 samples (before IVR administration); minimal to mild submucosal and/or intraepithelial eosinophil infiltrates were evident by Day 3. Estrogen has been reported as a strong inducer of eosinophil migration into the female reproductive tract, including the vagina [44,45,46]. While there were minor increases in the incidence and severity of mononuclear cells (macrophages, lymphocytes, plasma cells) in the submucosa, their presence at day 0, combined with the wide range of incidence and severity throughout the study, suggests that any differences were the result of normal variation (particularly as there is a high incidence of inflammatory cell infiltrates reported in the vaginas of sheep [47]). 

Ultimately, the microscopic findings between the NuvaRing cohort (Figure 8) and the CLIP_HIGH_ IVR cohort (Figure 9) at the timepoints for which biopsies were available for both (Day 0–Day 21) were not meaningfully different and were consistent with the pharmacologic effects of estrogen, despite the differences in IVR material and an increased hormone dose in the CLIP_HIGH_ IVRs. Thus, increasing the hormone dose to support the development of a 90-day IVR should not promote toxicity in the sheep model. In addition, although the CLIP_LOW_ IVRs had a similar dose to NuvaRing, the histological profiles cannot be compared due to the presence of an antiretroviral in the CLIP_LOW_ IVR when previously administered to sheep [12]. However, there were no adverse IVR-related findings with the CLIP_LOW_ IVR despite the presence of an antiretroviral, as previously described [12]. 

## 4. Conclusions

In this study, we investigated the effect of IVR manufacturing (IM vs. 3D-printing) on ENG and EE in vitro release, in vivo pharmacokinetics and safety in sheep [26]. Here, we compared our 3D-printed CLIP_LOW_ IVR to NuvaRing, the well-established commercially available monthly IVR for contraception. Our results demonstrated that NuvaRing’s releases was slightly greater than the CLIP_LOW_ IVR. This is the first report to our knowledge of ENG and EE levels in plasma, vaginal tissue and vaginal fluid from NuvaRing when administered to sheep [26]. This determined a preclinical contraceptive benchmark for the vaginal delivery of these hormonal steroids in the ovine model, which can ultimately be used for efficient optimization in the preclinical development of vaginal delivery systems containing ENG or EE. Leveraging these effective contraceptive levels from NuvaRing in sheep, we optimized our ENG/EE loadings to develop a long-acting CLIP_HIGH_ IVR and showed systemic and local drug levels greater than the levels achieved with NuvaRing over 90 days in vivo with contraceptive reversibility based on the rapid clearance of ENG and EE after IVR removal [26]. There were no safety concerns after the evaluation of CLIP_HIGH_ IVRs for 92 days in sheep. 

Although our study demonstrated promising results, there are limitations to the work described in this manuscript. For example, it is important to note that in vitro release studies with NuvaRing were only performed with *n* = 1. Although NuvaRing is a well-established commercial product with expected low variability and observed variability in sheep studies was low (*n* = 4), additional studies should be performed to confirm low variability in vitro with a greater sample size. Another limitation is the use of the ovine model for in vivo studies. Although sheep represent a relevant animal model for studying vaginally delivered technologies, due to their similarities in body mass and size of the vaginal canal compared to humans, sheep elicit a vaginal pH of ~7, whereas the vaginal pH in humans is ~4. This difference in pH can impact release of drugs that elicit a strong pH dependence, which would decrease the translatability from sheep data to expected results in humans. ENG and EE do not have pH-dependent drug release; thus, the sheep model is translatable here. Finally, all CLIP IVRs generated in this manuscript were fabricated using a small-scale 3D-printer (16 rings printed per batch) and a lab scale drug loading process post-fabrication. However, larger models of the Carbon 3D-printers are readily available and well-established (M2 and L1, Carbon, Inc.) and GMP manufacturing using the M2 printers is already established, which makes large scale manufacturing feasible in the future. We also acknowledge the novelty of the manufacturing process and, as such, the need for full biocompatibility and extractable and leachable assessment to confirm that the final device form, following fabrication and drug loading, does not present any new impurities, degradants or residual solvents that might create a different safety profile to existing silicone-based polymer vaginal drug-delivery systems.

In the future, we plan to combine the CLIP_HIGH_ IVRs described in this manuscript with an antiretroviral to develop a long-acting (≥90 days) MPT IVR for the prevention of HIV and unintended pregnancy. Additionally, we plan to further optimize the CLIP_HIGH_ IVR by incorporating an IVR design to further fine-tune the drug release and achieve precise target drug release rates to be sustained for 90 days or longer with 100% drug release upon completion prior to subsequent doses. Once the IVR design and inclusion of an antiretroviral in CLIP_HIGH_ IVR has been optimized, we plan to perform stability studies to define storage conditions and shelf-life, and conduct in vivo studies to assess PK, safety and efficacy in sheep and rhesus macaque models.

Collectively, results from this study provided a comprehensive comparison of ENG and EE release from a 3D-printed IVR and NuvaRing, determined a contraceptive ENG/EE PK benchmark in the ovine model and optimized a long-acting CLIP_HIGH_ IVR to be further developed as an MPT IVR in future studies.

## Data Availability

The data supporting the findings of the study are available from the corresponding author, upon reasonable request.

## Supplementary Materials

The following supporting information can be downloaded at: https://www.mdpi.com/article/10.3390/pharmaceutics16081030/s1, Supplementary Figure S1. Individual replicates of ENG/EE sheep PK for CLIP_LOW_ IVR and NuvaRing. Four (*n* = 4) female sheep were administered CLIP_LOW_ IVR or NuvaRing for 14 or 21 days, respectively. ENG/EE concentration in plasma for (A) ENG and (B) EE. ENG/EE concentration in vaginal tissue for (C) ENG and (D) EE. ENG/EE concentration in vaginal fluids for (E) ENG and (F) EE. Lower limits of quantification (LLOQs) of ENG in plasma, vaginal tissue and vaginal fluid are 0.2 ng/mL, 17 ng/g and 0.215 ng/swab, respectively. LLOQs of EE in plasma, vaginal tissue and vaginal fluid are 0.005 ng/mL, 4.2 ng/g and 0.108 ng/swab, respectively. Samples that are below the limit of quantification are represented as LLOQ/2. Supplementary Figure S2. Individual replicates of ENG/EE sheep PK from CLIPHIGH IVR. Three (*n* = 3) female sheep were administered the high hormone dose (40 mg ENG and 10 mg EE) CLIPHIGH IVR for the entire 92-day duration. ENG/EE concentrations in (A) plasma, (B) vaginal tissue and (C) vaginal fluids. Lower limits of quantification (LLOQs) of ENG in plasma, vaginal tissue and vaginal fluid are 0.2 ng/mL, 17 ng/g and 0.215 ng/swab, respectively. LLOQs of EE in plasma, vaginal tissue and vaginal fluid are 0.005 ng/mL, 4.2 ng/g and 0.108 ng/swab, respectively. Samples that are below the limit of quantification are represented as LLOQ/2. Supplementary Figure S3. Individual replicates of ENG/EE levels in plasma, vaginal fluids and vaginal tissue from CLIP_HIGH_ IVR before and after IVR removal. Six (*n* = 6) female sheep were administered CLIP_HIGH_ IVR. Individual replicates of sheep that received CLIP_HIGH_ IVR removed at 30 days post-IVR administration in (A) plasma, (B) vaginal fluid and (C) vaginal tissue. Individual replicates of sheep that received CLIP_HIGH_ IVR removed at 60 days post-IVR administration in (D) plasma, (E) vaginal fluid and (F) vaginal tissue. Individual replicates of sheep that received CLIP_HIGH_ IVR removed at 92 days post-IVR administration in (G) plasma, (H) vaginal tissue and (I) vaginal fluids. Data from all sheep (*n* = 6) were pooled together for timepoints when IVR was in the body. At day 30 post-IVR administration, *n* = 2 IVRs were removed from sheep (A–C) and at days 60 and 92 post-IVR administration, *n* = 3 IVRs were removed (D–I). Solid lines indicate when IVRs were in the body and dashed lines indicate IVR removal. Lower limits of quantification (LLOQ) of ENG in plasma, vaginal tissue and vaginal fluid are 0.1 ng/mL, 5.11 ng/g and 0.215 ng/swab, respectively. LLOQs of EE in plasma, vaginal tissue and vaginal fluid are 0.005 ng/mL, 2.55 ng/g and 0.054 ng/swab, respectively. Samples that are below the limit of quantification are represented as LLOQ/2. Supplementary Figure S4. Histology scores for individual sheep administered NuvaRing. Individual histological scores from four (*n* = 4) female sheep that were administered NuvaRing for 21 days. Day 0 represents the baseline with no IVR use. Supplementary Figure S5. Histology scores for individual sheep administered CLIP_HIGH_ IVR for 28 days. Individual histological scores from two (*n* = 2) female sheep that were administered the CLIP_HIGH_ IVR for 28 days. Day 0 represents the baseline with no IVR use. Supplementary Figure S6. Histology scores for individual sheep administered CLIP_HIGH_ IVR for 60 days. Individual histological scores from three (*n* = 3) female sheep that were administered the CLIP_HIGH_ IVR for 60 days. Day 0 represents the baseline with no IVR use. Supplementary Figure S7. Histology scores for individual sheep administered CLIP_HIGH_ IVR for 92 days. Individual histological scores from three (*n* = 3) female sheep that were administered the CLIP_HIGH_ IVR for 92 days. Day 0 represents the baseline with no IVR use.
