# Supplementary material for: Next-Generation Contraceptive Intravaginal Ring: Comparison of Etonogestrel and Ethinyl Estradiol In Vitro and In Vivo Release from 3D-Printed Intravaginal Ring and NuvaRing
Source: Pharmaceutics. 2024 Aug 2;16(8):1030. doi: 10.3390/pharmaceutics16081030 (PMC11359822; doi:10.3390/pharmaceutics16081030)
Supplement: Supplementary file 1 [file pharmaceutics-16-01030-s001.zip › pharmaceutics-3094506-supplementary.pdf]

Supplementary Materials

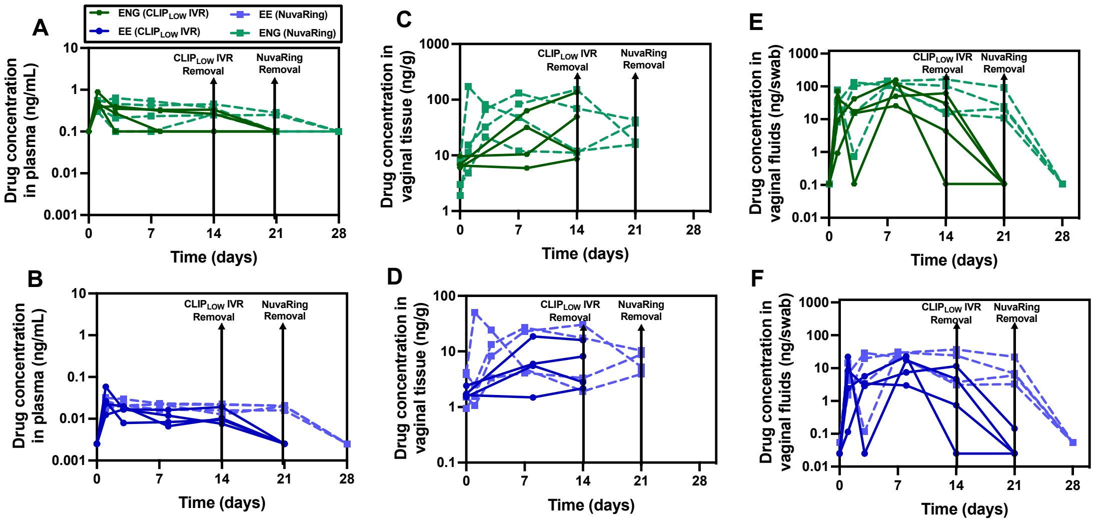

Supplementary Figure S1. Individual replicates of ENG/EE sheep PK for CLIPLow IVR and NuvaRing. Four (n=4) female sheep were administered CLIPLow IVR or NuvaRing for 14 or 21 days, respectively. ENG/EE concentration in plasma for (A) ENG and (B) EE. ENG/EE concentration in vaginal tissue for (C) ENG and (D) EE. ENG/EE concentration in vaginal fluids for (E) ENG and (F) EE. Lower limit of quantification (LLOQ) of ENG in plasma, vaginal tissue, and vaginal fluid is 0.2 ng/mL, 17 ng/g, and 0.215 ng/swab, respectively. LLOQ of EE in plasma, vaginal tissue, and vaginal fluid is 0.005 ng/mL, 4.2 ng/g, and 0.108 ng/swab, respectively. Samples that were below the limit of quantification were represented as LLOQ/2.

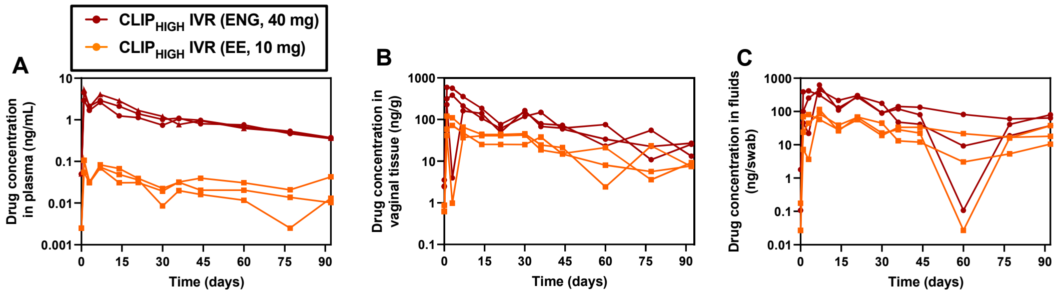

Supplementary Figure S2. Individual replicates of ENG/EE sheep PK from CLIPHigh IVR. Three (n=3) female sheep were administered the high hormone dose (40 mg ENG and 10 mg EE) CLIPHigh IVR for the entire 92-day duration. ENG/EE concentrations in (A) plasma, (B) vaginal tissue, and (C) vaginal fluids. Lower limit of quantification (LLOQ) of ENG in plasma, vaginal tissue, and vaginal fluid is 0.2 ng/mL, 17 ng/g, and 0.215 ng/swab, respectively. LLOQ of EE in plasma, vaginal tissue, and vaginal fluid is 0.005 ng/mL, 4.2 ng/g, and 0.108 ng/swab, respectively. Samples that were below the limit of quantification were represented as LLOQ/2.

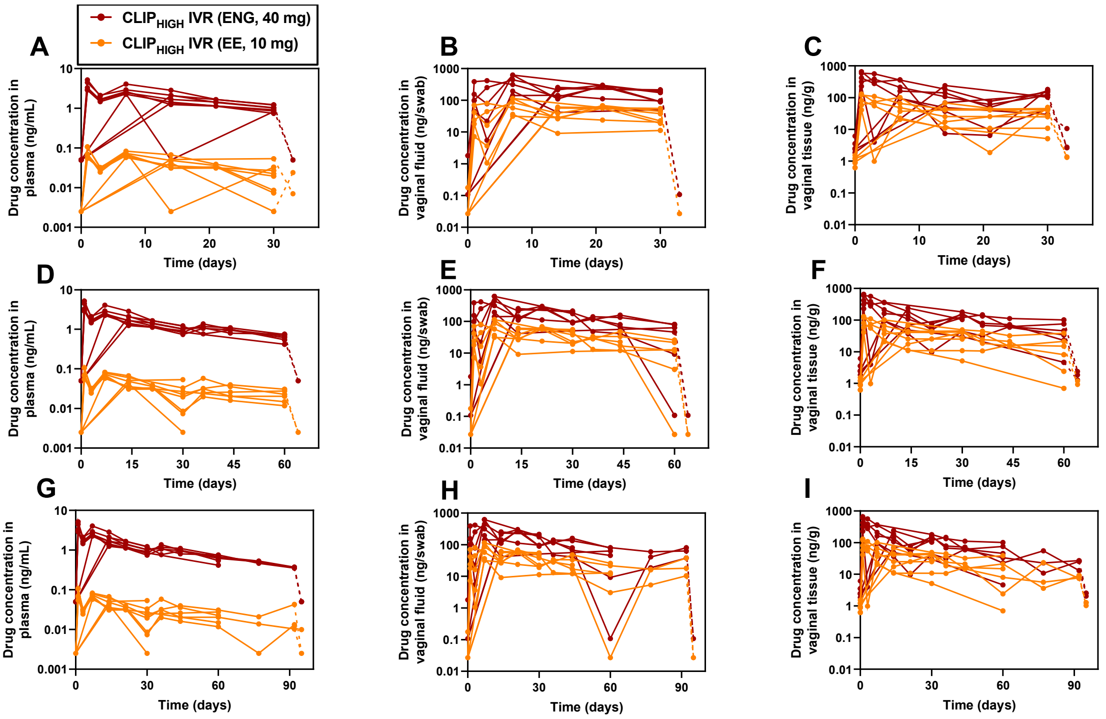

Supplementary Figure S3. Individual replicates of ENG/EE levels in plasma, vaginal fluids, and vaginal tissue from CLIPHIGH IVR before and after IVR removal. Six (n=6) female sheep were administered CLIPHIGH IVR. Individual replicates of sheep that received CLIPHIGH IVR and removed at 30-days post-IVR administration in (A) plasma, (B) vaginal fluid, and (C) vaginal tissue. Individual replicates of sheep that received CLIPHIGH IVR and removed at 60-days post-IVR administration in (D) plasma, (E) vaginal fluid, and (F) vaginal tissue. Individual replicates of sheep that received CLIPHIGH IVR and removed at 92-days post-IVR administration in (G) plasma, (H) vaginal tissue, and (I) vaginal fluids. Data from all sheep (n=6) were pooled together for timepoints when IVR was in the body. At day 30 post-IVR administration, n=2 IVRs were removed from sheep (A-C) and at day 60 and 92 post-IVR administration, n=3 IVRs were removed (D-I). Solid lines indicate when IVRs were in the body and dashed lines indicate IVR removal. Lower limit of quantification (LLOQ) of ENG in plasma, vaginal tissue, and vaginal fluid is 0.1 ng/mL, 5.11 ng/g, and 0.215 ng/swab, respectively. LLOQ of EE in plasma, vaginal tissue, and vaginal fluid is 0.005 ng/mL, 2.55 ng/g, and 0.054 ng/swab, respectively. Samples that were below the limit of quantification were represented as LLOQ/2.

Sheep NuvaRing (21 days)	Days (IVR wear)					
**Sheep ID 1063**	**0**	**1**	**3**	**7**	**14†**	**21**
**Epithelium**	**Score**					
Hyperplasia, epithelial	0	0	0	1	0	0
Atrophy, epithelial	1	0	0	0	0	0
Inflammatory cells, intraepithelial	1	1	1	2*	1	1
Globular cells (Mott Cells)	0	0	0	0	NA	0
Edema, intraepithelial	0	0	0	1	1	0
Edema, interepithelial	2	1	1	1	1	0
Apoptosis, individual cells	0	0	0	0	0	0
Parakeratosis	0	1	1	0	1	1
**Submucosa**	**Score**					
Infiltrates, PMNs	0	0	0	0	NA	0
Infiltrates, eosinophils	0	1	1	2	NA	0
Infiltrates, mononuclear	1	1	1	2	NA	1
**Perivascular**, mononuclear	0	0	0	1	0	1

Sheep NuvaRing (21 days)	Days (IVR wear)					
**Sheep ID 1100**	**0**	**1**	**3**	**7†**	**14**	**21**
**Epithelium**	**Score**					
Hyperplasia, epithelial	0	0	0	0	0	0
Atrophy, epithelial	0	0	0	1	0	1
Inflammatory cells, intraepithelial	1	1	2	1	1	1
Globular cells (Mott Cells)	0	0	0	0	0	0
Edema, intraepithelial	1	0	0	1	1	1
Edema, interepithelial	1	1	1	1	1	1
Apoptosis, individual cells	0	0	0	0	0	0
Parakeratosis	0	1	1	1	2	2
**Submucosa**	**Score**					
Infiltrates, PMNs	0	0	0	0	0	0
Infiltrates, eosinophils	0	0	0	0	0	2
Infiltrates, mononuclear	0	1	2	2	2	2
**Perivascular**, mononuclear	0	0	0	1	1	2

Sheep NuvaRing (21 days)	Days (IVR wear)					
**Sheep ID 1107**	**0**	**1**	**3**	**7**	**14†**	**21**
**Epithelium**	**Score**					
Hyperplasia, epithelial	0	0	No tissue	1	0	0
Atrophy, epithelial	0	0		0	0	1
Inflammatory cells, intraepithelial	0	1		2	2	1
Globular cells (Mott Cells)	0	0		0	0	0
Edema, intraepithelial	1	0		1	1	1
Edema, interepithelial	1	0		1	1	0
Apoptosis, individual cells	0	1		0	0	0
Parakeratosis	0	0		1	0	2
**Submucosa**	**Score**					
Infiltrates, PMNs	0	0	No tissue	0	0	0
Infiltrates, eosinophils	0	0		0	1	1
Infiltrates, mononuclear	1	1		1	1	1
**Perivascular**, mononuclear	0	0		1	0	0

Sheep NuvaRing (21 days)	Days (IVR wear)					
**Sheep ID 1108**	**0**	**1**	**3**	**7**	**14**	**21**
**Epithelium**	**Score**					
Hyperplasia, epithelial	0	0	1	0	0	0
Atrophy, epithelial	0	0	0	0	2	2
Inflammatory cells, intraepithelial	1	1	1	1*	1	1
Globular cells (Mott Cells)	0	0	0	0	0	0
Edema, intraepithelial	1	0	1	1	0	0
Edema, interepithelial	1	1	1	1	0	0
Apoptosis, individual cells	1	0	0	0	0	0
Parakeratosis	0	2	2	1	2	2
**Submucosa**	**Score**					
Infiltrates, PMNs	0	0	0	0	0	0
Infiltrates, eosinophils	0	0	1	1	0	0
Infiltrates, mononuclear	1	0	1	1	1	1
**Perivascular**, mononuclear	0	0	0	1	0	0

* transmigrating cells appear to be eosinophils
† moderate damage to biopsy hinders interpretation

Supplementary Figure S4. Histology scores for individual sheep administered NuvaRing. Individual histological scores from four (n=4) female sheep that were administered NuvaRing for 21 days. Day 0 represents the baseline with no IVR wear.

Sheep Received CLIP ENG/EE IVR (28 days)	Days (IVR wear)			Days (Post-IVR removal)	
**Sheep ID 1045**	**0**	**13**	**28**	**3**	**7**
**Epithelium**	**Score**				
Hyperplasia, epithelial	1	0	0	0	0
Atrophy, epithelial	0	0	1	0	0
Inflammatory cells, intraepithelial	0	1	0	1	0
Globular cells (Mott Cells)	0	0	0	0	0
Edema, intraepithelial	0	0	0	0	1
Edema, interepithelial	1	0	0	0	1
Apoptosis, individual cells	0	0	0	0	1
Parakeratosis	1	1	1	1	0
**Submucosa**	**Score**				
Infiltrates, PMNs	1	1	0	1	0
Infiltrates, eosinophils	0	0	0	0	0
Infiltrates, mononuclear	2	1	1	2	0

Sheep Received CLIP ENG/EE IVR (28 days)	Days (IVR wear)			Days (Post-IVR removal)	
**Sheep ID 1190**	**0**	**13**	**28**	**3**	**7**
**Epithelium**	**Score**				
Hyperplasia, epithelial	0	0	0	0	0
Atrophy, epithelial	1	0	0	0	0
Inflammatory cells, intraepithelial	1	1	2	0	1
Globular cells (Mott Cells)	0	1	0	0	0
Edema, intraepithelial	2	0	1	0	0
Edema, interepithelial	1	1	2	0	0
Apoptosis, individual cells	1	0	0	0	1
Parakeratosis	0	1	0	0	0
**Submucosa**	**Score**				
Infiltrates, PMNs	1	1	0	0	0
Infiltrates, eosinophils	0	1	1	0	1
Infiltrates, mononuclear	1	2	1	1	1

Supplementary Figure S5. Histology scores for individual sheep administered CLIPHIGH IVR for 28 days. Individual histological scores from two (n=2) female sheep that were administered the CLIPHIGH IVR for 28 days. Day 0 represents the baseline with no IVR wear.

Sheep Received CLIP ENG/EE IVR (60 days)	Days (IVR wear)										Days (Post-IVR removal)	
**Sheep ID 1045**	**0**	**1**	**3**	**7**	**14**	**21**	**30**	**36**	**44**	**60**	**3**	**7**
**Epithelium**	**Score**											
Hyperplasia, epithelial	0	0	0	0	0	0	0	0	0	0	1	1
Atrophy, epithelial	2	0	0	2	2	2	2	2	0	1	0	0
Inflammatory cells, intraepithelial	2	1	1	1	1	0	0	0	1	1	1	1
Globular cells (Mott Cells)	1	0	0	0	0	0	0	0	0	0	1	0
Edema, intraepithelial	0	1	0	1	0	1	1	0	1	1	0	0
Edema, interepithelial	0	0	0	1	0	1	0	0	0	0	0	1
Apoptosis, individual cells	0	0	0	0	0	0	0	0	0	0	0	1
Parakeratosis	0	1	1	1	2	1	1	0	1	1	1	0
**Submucosa**	**Score**											
Infiltrates, PMNs	1	1	0	1	1	0	0	1	0	1	1	0
Infiltrates, eosinophils	0	0	0	0	0	1	0	0	0	0	0	0
Infiltrates, mononuclear	2	1	0	2	2	1	1	2	0	1	1	0
**Perivascular**, mononuclear	0	0	0	1	1	0	0	0	0	0	0	0

Sheep Received CLIP ENG/EE IVR (60 days)	Days (IVR wear)								Days (Post-IVR removal)	
**Sheep ID 1063**	**0**		**6**	**14**		**30**		**47**	**62**	**3**	**7**
**Epithelium**	**Score**									
Hyperplasia, epithelial	0		0	0		0		1	0	0	0
Atrophy, epithelial	1		0	0		1		0	0	0	0
Inflammatory cells, intraepithelial	2		2	1		1		2*	2*	2*	2*
Globular cells (Mott Cells)	0		0	0		0		0	0	0	0
Edema, intraepithelial	0		1	0		0		0	0	0	1
Edema, interepithelial	0		0	1		1		2	0	1	0
Apoptosis, individual cells	0		0	0		0		0	0	0	0
Parakeratosis	1		0	0		2		1	0	0	1
**Submucosa**	**Score**									
Infiltrates, PMNs	1		0	0		1		0	0	1	0
Infiltrates, eosinophils	0		0	0		0		2	2	0	1
Infiltrates, mononuclear	1		2	2		1		0	1	1	1
**Perivascular**, mononuclear	0		0	0		1		0	0	0	0

Sheep Received CLIP ENG/EE IVR (60 days)	Days (IVR wear)									
**Sheep ID 1090**	**0**	**1**	**3**	**7**	**14**	**21**	**30**	**36**	**44**	**60**
**Epithelium**	**Score**									
Hyperplasia, epithelial	0	1	1	0	1	1	0	0	1	0
Atrophy, epithelial	1	0	0	1	0	0	0	0	0	1
Inflammatory cells, intraepithelial	2	1	1	1	2	1	1	1	2	1
Globular cells (Mott Cells)	0	0	0	0	0	0	0	0	0	0
Edema, intraepithelial	0	0	1	1	1	1	2	1	0	0
Edema, interepithelial	0	0	0	0	1	1	0	1	0	1
Apoptosis, individual cells	2	1	0	0	0	0	0	0	0	0
Parakeratosis	0	1	1	1	1	0	0	0	1	0
**Submucosa**	**Score**									
Infiltrates, PMNs	1	1	1	1	1	0	0	0	1	0
Infiltrates, eosinophils	0	0	0	0	1	0	0	2	0	1
Infiltrates, mononuclear	1	1	1	1	2	0	1	2	2	1
**Perivascular**, mononuclear	0	0	0	1	0	0	0	0	1	1

*Intraepithelial cells are mostly eosinophils

Supplementary Figure S6. Histology scores for individual sheep administered CLIPHIGH IVR for 60 days. Individual histological scores from three (n=3) female sheep that were administered the CLIPHIGH IVR for 60 days. Day 0 represents the baseline with no IVR wear.

Sheep Received CLIP ENG/EE IVR (90 days)	Days (IVR wear)												Days (Post-IVR removal)	
**Sheep ID 1100**	**0**	**1**	**3**	**7**	**14**	**21**	**30**	**36**	**44**	**60**	**77**	**92**	**3**	**7***
**Epithelium**	**Score**													
Hyperplasia, epithelial	0	0	0	0	0	0	0	0	0	0	0	0		0
Atrophy, epithelial	0	0	0	0	0	1	0	1	0	0	0	0		0
Inflammatory cells, intraepithelial	2	0	1	2	1	1	1	1	1	1	1	1		1
Globular cells (Mott Cells)	0	0	0	0	0	0	0	0	0	0	0	0	***	1
Edema, intraepithelial	0	0	0	0	1	1	0	1	0	1	2	2		1
Edema, interepithelial	1	0	0	1	1	2	0	0	0	0	2	2		1
Apoptosis, individual cells	0	0	0	0	0	0	0	0	0	0	0	0		1
Parakeratosis	0	1	2	2	1	1	1	1	1	1	2	2		0
**Submucosa**	**Score**													
Infiltrates, PMNs	1	1	1	2	2	0	0	1	0	0	0	0	0	0
Infiltrates, eosinophils	0	0	0	0	0	0	1	0	0	1	2	2	0	1
Infiltrates, mononuclear	1	1	1	2	2	2	2	1	1	1	1	1	0	2
**Perivascular**, mononuclear	0	0	0	0	2	0	0	0	2	0	0	0	0	0

Sheep Received CLIP ENG/EE IVR (90 days)	Days (IVR wear)												Days (Post-IVR removal)	
**Sheep ID 1107**	**0**	**1**	**3**	**7**	**14**	**21**	**30**	**36**	**44**	**60**	**77**	**92**	**3**	**7***
**Epithelium**	**Score**													
Hyperplasia, epithelial	0	0	0	0	0	0	0	0	0	0	0	0	0	0
Atrophy, epithelial	0	0	1	0	0	0	1	2	0	0	1	1	1	1
Inflammatory cells, intraepithelial	1	1	1	2	1	1	1	1	1	1	2	1	1	1
Globular cells (Mott Cells)	0	0	0	0	0	0	0	0	0	0	0	0	0	2
Edema, intraepithelial	0	0	0	1	0	0	1	0	0	1	0	0	0	0
Edema, interepithelial	0	0	0	1	1	1	0	0	1	0	0	0	0	0
Apoptosis, individual cells	0	0	0	0	0	0	0	0	0	0	0	0	0	0
Parakeratosis	0	0	1	1	2	0	1	1	0	0	1	2	0	0
**Submucosa**	**Score**													
Infiltrates, PMNs	0	1	1	1	1	0	1	0	0	0	0	1	0	0
Infiltrates, eosinophils	0	0	0	0	0	0	0	0	1	2	2	0	0	0
Infiltrates, mononuclear	0	1	1	1	1	0	2	1	2	2	2	1	3	2
**Perivascular**, mononuclear	0	1	0	0	0	0	0	0	0	2	0	0	3	0

Sheep Received CLIP ENG/EE IVR (90 days)	Days (IVR wear)												Days (Post-IVR removal)	
**Sheep ID 1172**	**0**	**1**	**3**	**7**	**14**	**21**	**30**	**36**	**44**	**60**	**77**	**92**	**3**	**7**
**Epithelium**	**Score**													
Hyperplasia, epithelial	0	1	0	0	0	0	0	0	0	0	0	0	0	0
Atrophy, epithelial	0	0	1	2	1	2	0	2	1	1	1	0	0	1
Inflammatory cells, intraepithelial	0	1	2*	2*	1	1	1	1	1	1	1	1	1	1
Globular cells (Mott Cells)	0	0	0	0	0	0	0	0	0	0	0	0	0	0
Edema, intraepithelial	0	0	1	0	0	0	0	0	0	0	0	0	0	0
Edema, interepithelial	0	0	1	1	0	1	0	0	0	0	0	1	0	0
Apoptosis, individual cells	0	0	0	0	0	0	0	0	0	0	0	0	1	0
Parakeratosis	0	1	1	2	1	0	0	0	1	0	0	0	0	0
**Submucosa**	**Score**													
Infiltrates, PMNs	0	0	1	1	0	0	1	1	0	1	0	0	0	0
Infiltrates, eosinophils	0	1	2	2	1	1	0	0	1	0	2	2	1	0
Infiltrates, mononuclear	0	2	2	3	3	3	2	2	3	2	2	2	1	2
**Perivascular**, mononuclear	0	0	0	0	0	0	0	2	0	0	0	0	0	0

*Intraepithelial cells are mostly eosinophils
***inconclusive evaluation due to damage

Supplementary Figure S7. Histology scores for individual sheep administered CLIPHIGH IVR for 92 days. Individual histological scores from three (n=3) female sheep that were administered the CLIPHIGH IVR for 92 days. Day 0 represents the baseline with no IVR wear.
